# Supplementary material for: Roles of histone chaperone Nap1 and histone acetylation in regulating phase-separation of nucleosome arrays
Source: Nat Commun. 2025 Nov 27;16:10672. doi: 10.1038/s41467-025-65701-3 (PMC12661029; doi:10.1038/s41467-025-65701-3)
Supplement: Supplementary file 1 — Supplementary Information [file 41467_2025_65701_MOESM1_ESM.pdf]

Roles of histone chaperone Nap1 and histone acetylation in regulating phase-separation of nucleosome arrays

Supplementary Information

: Supplementary Figures 1 – 18 and Supplementary Table 1

Jia Gao[1], Hongyun Li[1], Song Tan[2], Ruobo Zhou[1,2], Tae-Hee Lee[1,*]

[1]Department of Chemistry and [2]Department of Biochemistry and Molecular Biology
The Pennsylvania State University, University Park, PA 16802

*Correspondence to: txl18@psu.edu

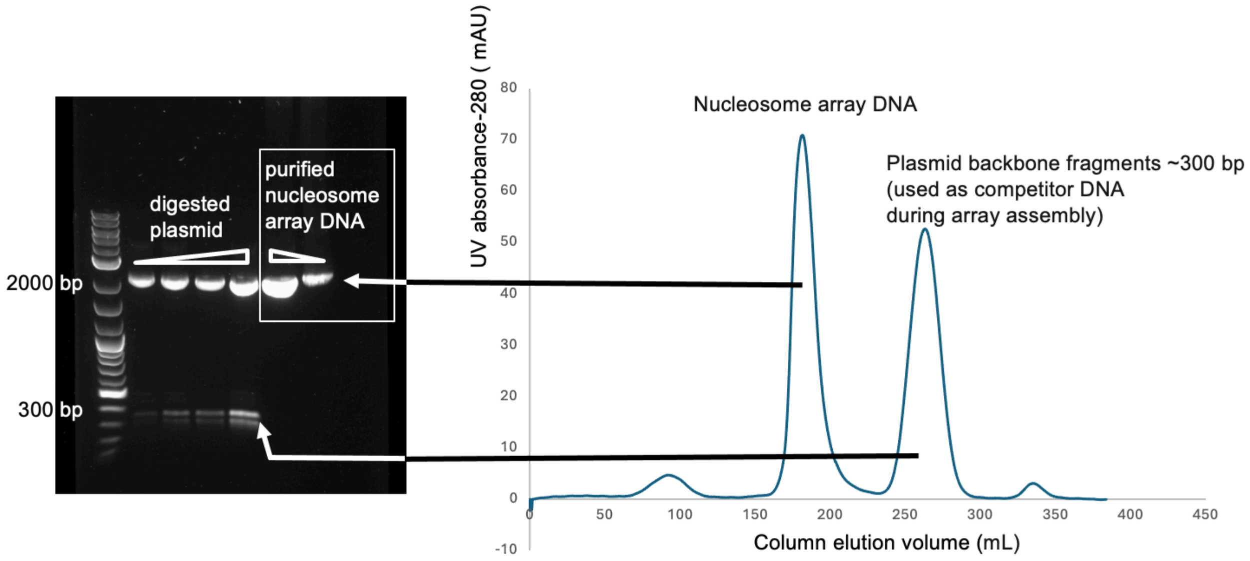

Supplementary Figure 1: A typical FPLC elution curve for DNA purification

A Sephacryl S-500HR column (26 mm inner diameter, ~900 mm bed height, 0.7 mL/min elution rate) in conjunction with an AKTA Pure 25M (Cytiva) FPLC system was used to purify nucleosome array DNA after digesting plasmids. The first main peak represents the nucleosome array DNA. The second main peak represents the other digestion product which is ~300 bp in length. This shorter fragment is used as competitor DNA to “absorb” the leftover histones after saturating the nucleosome array DNA. The digestion products before and after FPLC purification are shown on an agarose gel (left).

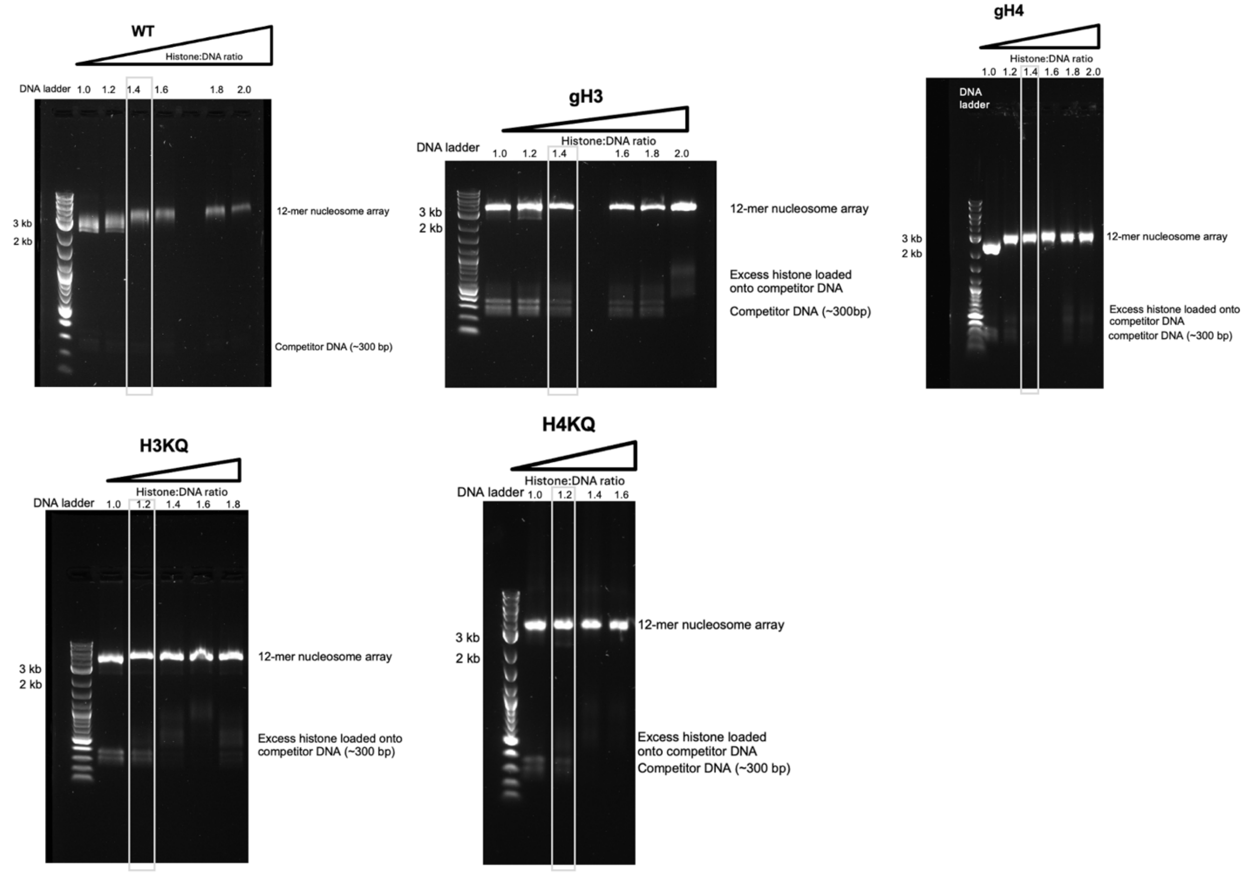

Supplementary Figure 2: Determination of DNA:histone ratio to saturate nucleosome array DNA for array preparation

DNA:histone titration was carried out to determine the DNA:histone ratio to saturate the nucleosome array DNA with histones for the 5 types of samples (WT, gH3, gH4, H3KQ, and H4KQ). A histone:DNA ratio (1:1.4 for WT, gH3, and gH4 arrays, and 1:1.2 for H3KQ and H4KQ arrays) that shows both array saturation (i.e., no further upper shift in the gel) and a sign of histone loading on the competitor DNA without precipitation was selected and used for array preparation via dialysis, purification with FPLC, and analysis with AUC (Supplementary Figure 3). At a proper DNA:histone ratio, competitor DNA starts forming aggregates of various sizes with randomly loaded excess histone after array DNA is saturated. The amounts of arrays and competitor DNA are constant across all lanes in each sample. The reason why the competitor DNA band becomes dimmer and shifts upward at a higher histone:DNA ratio is likely because the competitor DNA forms aggregates/precipitates of various sizes with randomly loaded excess histone after array DNA is saturated. For the WT arrays, a transmission electron microscopic analysis has also been carried out to further confirm the assembly (Supplementary Figure 4).

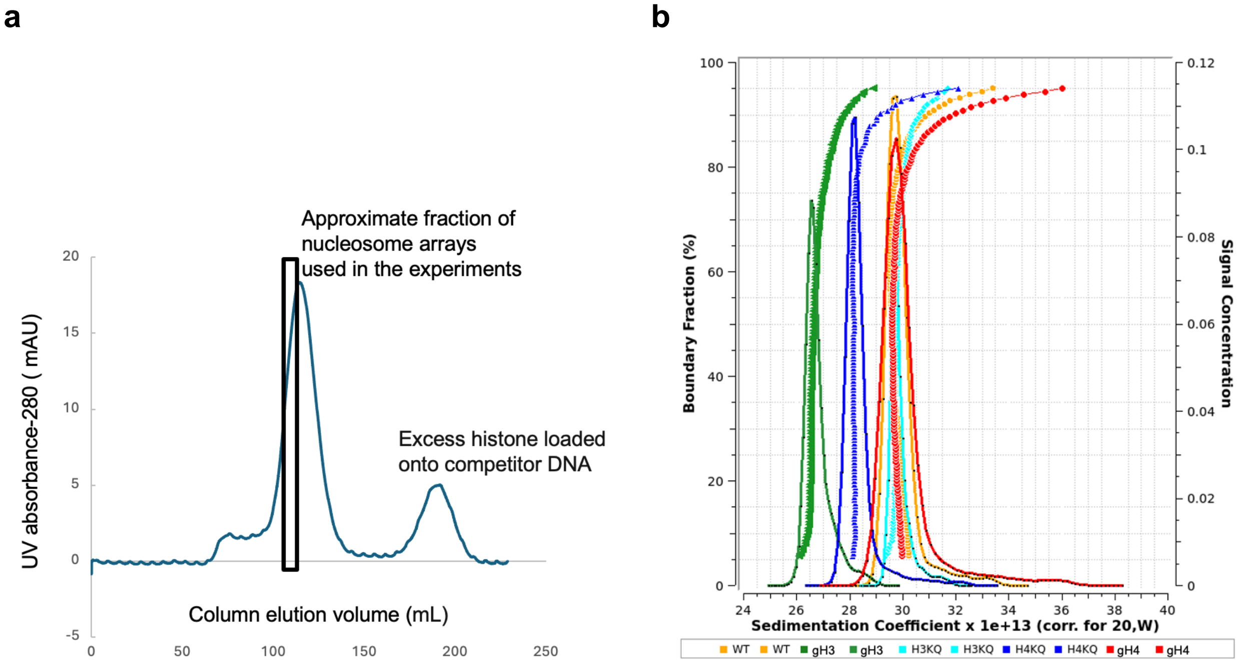

Supplementary Figure 3: A typical FPLC elution curve and sedimentation distributions of nucleosome arrays

a A Sephacryl S-500HR column (Cytiva, 26 mm inner diameter, ~900 mm bed height, 0.3 mL/min elution rate) in conjunction with an AKTA Pure 25M FPLC system (Cytiva) was used to fractionate nucleosome arrays after saturating the DNA with histones in the presence of ~300 bp competitor DNA. The saturating ratio of DNA:histone was determined by native gel analyses as shown in Supplementary Figure 2. Fractions in the first peak were analyzed using analytical ultracentrifugation (AUC). The highest molecular weight fraction with minimal over-saturated arrays was used for the experiments. Its approximate elution volume is marked on the curve. **b** The AUC data for the FPLC fraction used in the experiments are shown for the 5 types of nucleosome arrays (WT, gH3, gH4, H3KQ, and H4KQ). The data were obtained with Optima Ultra Multiwavelength Analytical Ultracentrifuge (Beckman Coulter, analysis with absorption at 280 nm) and analyzed with UltraScan III (van Holde-Weischet plots for $S_{20,w}$ distributions). For AUC analysis, array samples in a buffer containing 10 mM Tris-HCl (pH 7.5), 5 mM NaCl, 1mM EDTA, and 1 mM DTT (110 µL volume per sample, A_{260} UV absorbance value in the

range of 0.5 – 0.8) were added to AUC cells (3 mm) for a 6-hour run at 20,000 rpm and 20 ºC in an An-60 Ti rotor (Beckman Coulter). The rotor containing the sample was pre-equilibrated for 4 hours after pulling a full vacuum in the chamber. Radial absorbance scans at 280 nm were recorded every minute during the 6-hr run. The data was analyzed using UltraScan III. First, the radial intensity data were converted to pseudo-absorbance. The air-liquid meniscus was manually selected. The region of a sedimentation velocity distribution was also manually selected, which is usually contained in a 6.1 – 7.1 cm range. After discarding the first 5 - 10 radial scans (i.e., data from the first 5 - 10 minutes of sedimentation), the rest of the scans were fit with an S-value range between 1 and 10 at a resolution of 100 steps, and a frictional ratio range between 1 and 4 at a resolution of 64 steps. Fitting of time and radially invariant noises and 11 meniscus variations were also carried out to confirm proper selection of the meniscus. After selecting the best meniscus, another time and radially invariant noise fit was carried out using an iterative method. The data was analyzed with a Monte-Carlo genetic algorithm with 1 – 2 species per sample. The S-value and molecular weight distributions and the frictional ratio were extracted from the resulting pseudo-3-dimensional plots.

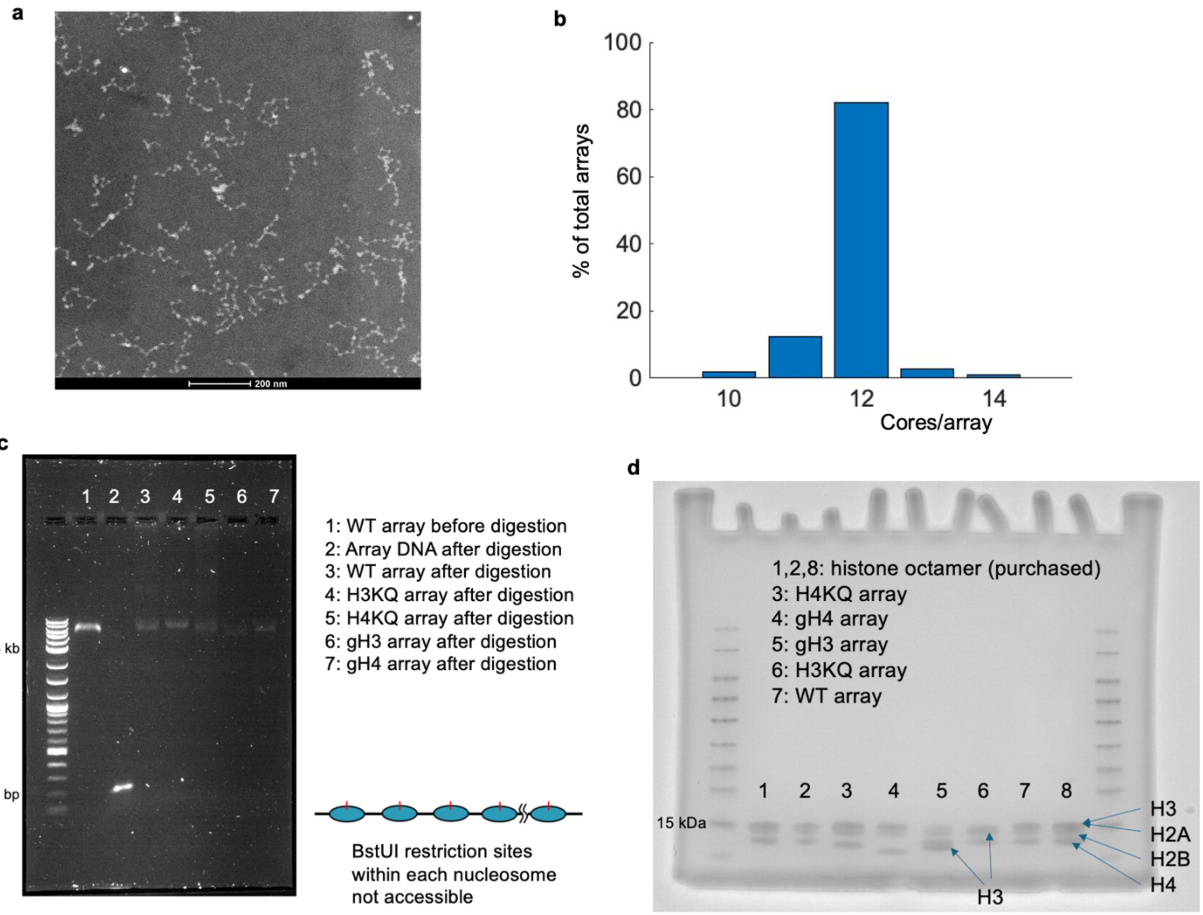

Supplementary Figure 4: A typical TEM image, a histogram, restriction enzyme digestion assay results, and SDS-PAGE results confirming the proper assembly of nucleosome arrays

a A transmission electron microscopy (TEM) image is shown to confirm nucleosome arrays made with unmodified histones (WT). To prepare for TEM imaging, nucleosome arrays were dialyzed against the HNE buffer (10 mM HEPES (pH 7.5), 5 mM NaCl, and 0.1 mM EDTA). An aliquot of 20 µL of dialyzed arrays at 15 – 20 µg/mL was cross-linked in 0.1 % glutaraldehyde for 5 hours at 4 °C, gently swirled every hour. The resulting sample was dialyzed against the HNE buffer at 4 °C. For imaging, the dialyzed sample (5 µL) was dropped on a carbon-coated EM grid after glow-discharging the surface. After 5-min incubation, the grid was dried by touching the sample drop with a torn edge of a filter paper. A 5 µL aliquot of 0.05 % uranyl acetate (staining reagent) was dropped on the grid and subsequently dried with another edge of

filter paper. The sample was first imaged at a fixed location with Talos F200C (Thermo Scientific) equipped with a 4k by 4k pixel CMOS camera, using the TEM mode at 200 kV illumination. After adjusting the focus to sharpen the image, the final image (2048 by 2115 pixels) of the area (0.6 by 0.6 μm^2 or 1.2 by 1.2 μm^2) was collected with the scanning TEM mode at 200 kV. **b** The number of histone cores was counted from all arrays that were visually resolvable. The histogram shows mostly 12-mer nucleosome arrays. The 11-mer arrays may have been over-counted due to the uncertainty associated with calling the number of histone cores based on visual inspection. **c** BstUI restriction enzyme digested array DNA and assembled arrays analyzed on a 1.5 % agarose gel confirm properly saturated arrays. **d** An SDS-PAGE gel image of nucleosome arrays shows approximately constant amounts of histones across all of the array samples, confirming properly assembled nucleosome arrays. Note that the H3 band is shifted in the gH3 and H3KQ arrays as is expected. A shift in the H4 band is also evident in the gH4 and H4KQ arrays. The samples were digested following the protocols provided by the manufacturer (New England Biolabs). Briefly, 50 µL of a sample containing 0.2 µg of arrays in 20 mM Tris-OAc (pH 7.9) buffer with 50 mM KOAc, 10 mM MgOAc, and 100 µg/mL albumin was incubated with 2 units of BstUI for 1 hour at 60 ^{o}C.

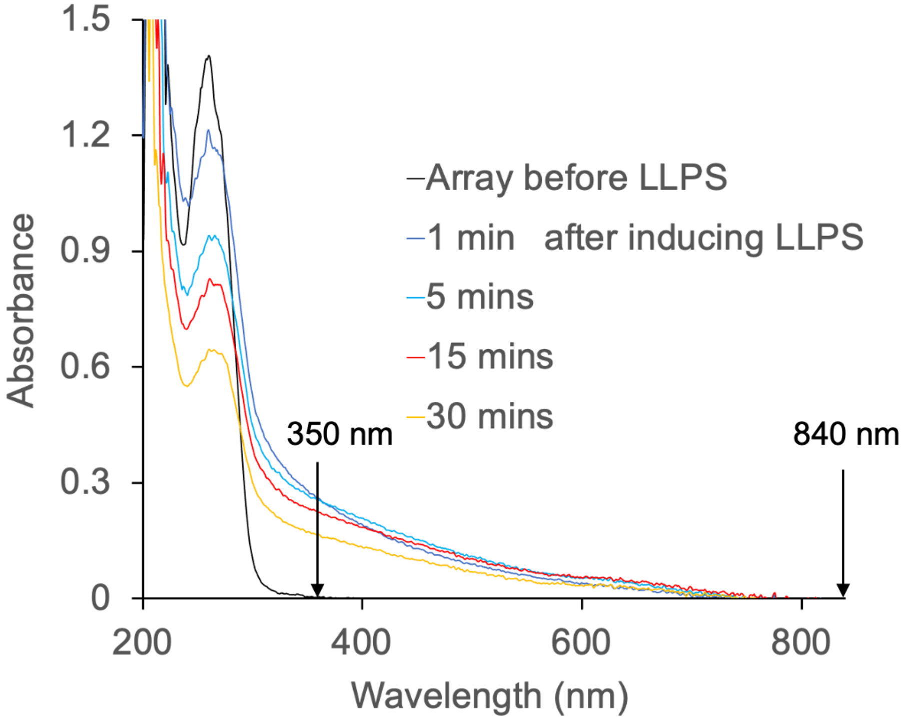

Supplementary Figure 5: UV-Vis spectra of nucleosome arrays (200 nM) mixed with 150 mM NaCl

Upon addition of NaCl to induce LLPS, nucleosome arrays form droplets that scatter light at wavelengths determined by their sizes which include the UV-Vis range. The scattering extent is measured as the absorbances in a UV-Vis spectrum. A time series of the UV-Vis spectrum (1, 5, 15, and 30 min after adding NaCl) reflects changes in the droplet size and population distribution that do not monotonically increase or decrease at a specific wavelength in 350 – 840 nm. The shortest wavelength for the measurements is set at 350 nm to avoid any interference from DNA or protein absorbance. The longest wavelength for the instrument (Nanodrop^{TM}, Thermofisher Scientific) is 840 nm. The integrated absorbances between 350 and 840 nm at the 30 min time point after LLPS induction were used to construct phase diagrams shown in Figs. 1 and 2. Of note, the UV-Vis spectra show generally decreasing absorbances between 350 – 840 nm after the 5 min time point, likely because droplets start fusing to form a smaller number of larger droplets with a few-µm diameter, which should result in less efficient scattering in the UV-Vis range.

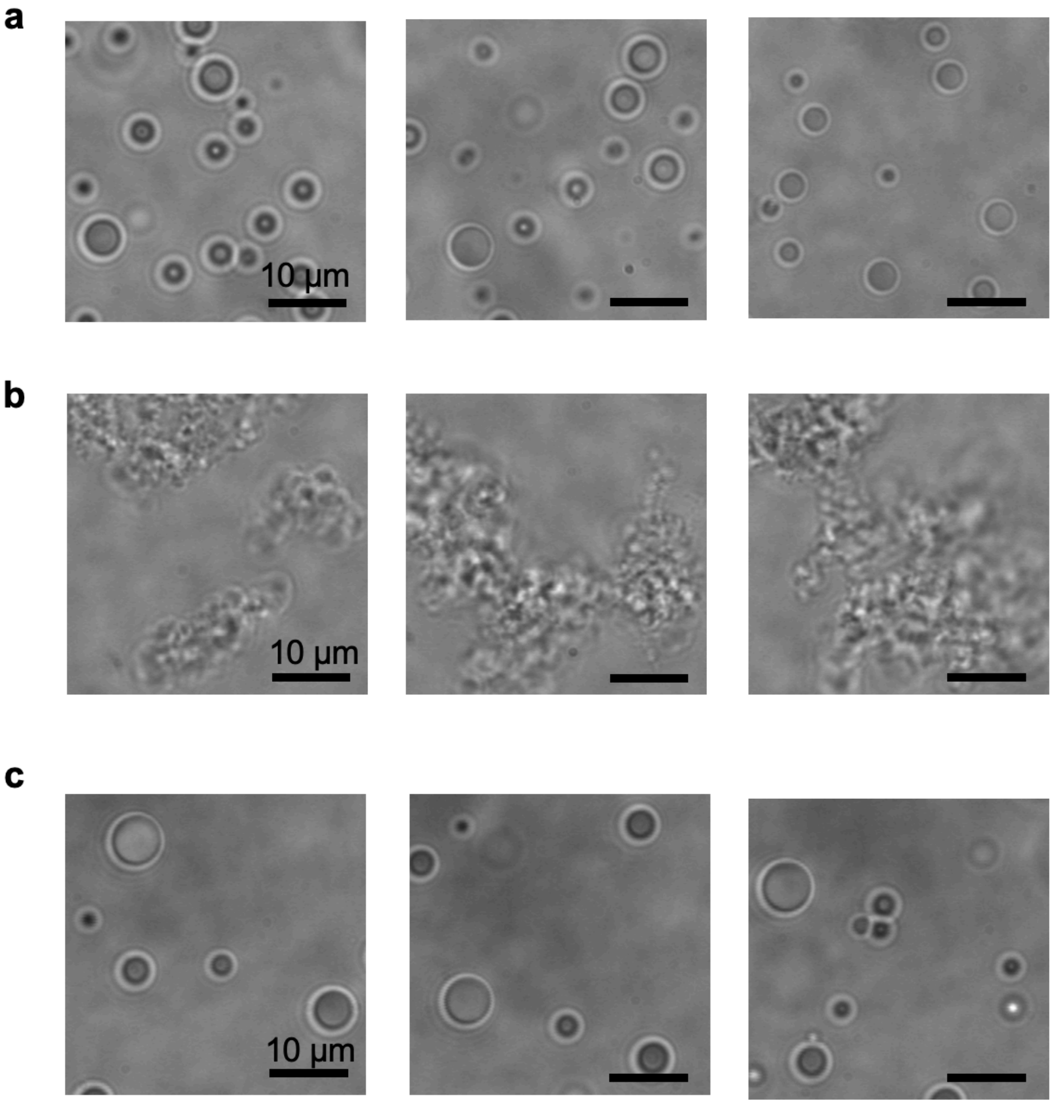

Supplementary Figure 6: Additional images of droplets and aggregates formed with H3KQ, gH3, and H3-acetylated arrays

Additional microscopic images of droplets and aggregates made of **a** H3KQ arrays, **b** gH3 arrays, and **c** WT arrays acetylated by Ada2/Ada3/Gcn5. All the arrays were at 200 nM concentration with 150 mM NaCl.

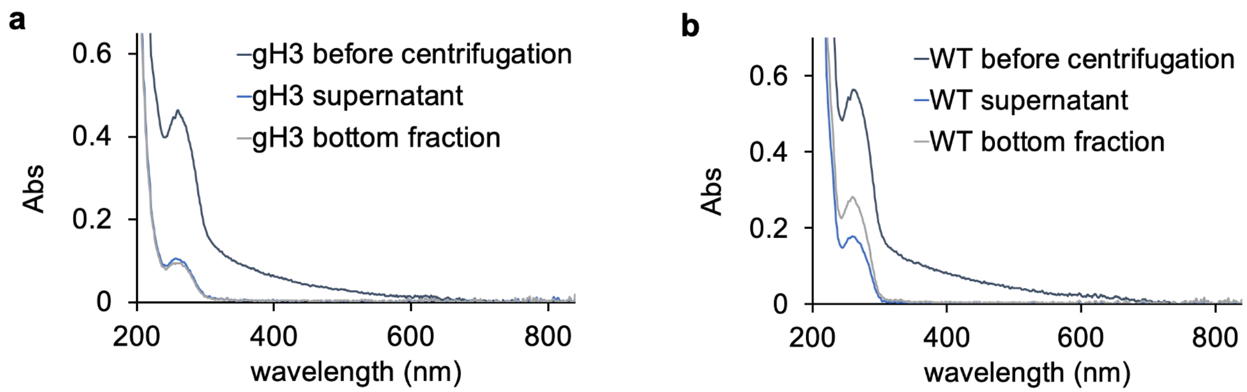

Supplementary Figure 7: UV-Vis spectra of nucleosome arrays from the supernatant and a bottom fraction after centrifugation of aggregates or droplets

Upon formation of aggregates or droplets (30 min induction), the UV-Vis absorbances were measured from the supernatant and a bottom fraction after centrifugation at 2,000 xg for 10 min. **a** In case of gH3 arrays, the supernatant and the bottom of the centrifuged sample show the same absorbance at 260 nm with no scattering in the range of 350 – 840 nm, supporting that all the aggregates were sedimented to a solid layer at the bottom of the tube that does not come off during the pipetting action. The same A260 values in the supernatant and the bottom fraction of the centrifuged sample confirm no existence of separate liquid phases, indicating that gH3 arrays does not form liquid-like droplets. **b** On the other hand, the same measurements with WT arrays clearly show a higher 260 nm absorbance from a bottom fraction than that from the supernatant with no scattering in the 350 – 840 nm range from either fraction. This result confirms that WT arrays form liquid droplets that sediment to a separate liquid layer at the bottom of a tube when centrifuged. Of note, the bottom layer of the WT arrays after centrifugation forms a sticky liquid-like film that cannot be easily transferred due to the stickiness. Because of this reason, only a small fraction of the film layer could be pipetted out, and consequently, the sample transferred to the UV-Vis instrument is contaminated with the supernatant. Nevertheless, no scattering in combination with increased DNA/protein absorbance upon sedimentation only with WT arrays confirm that WT arrays form liquid-like droplets while gH3 arrays form aggregates.

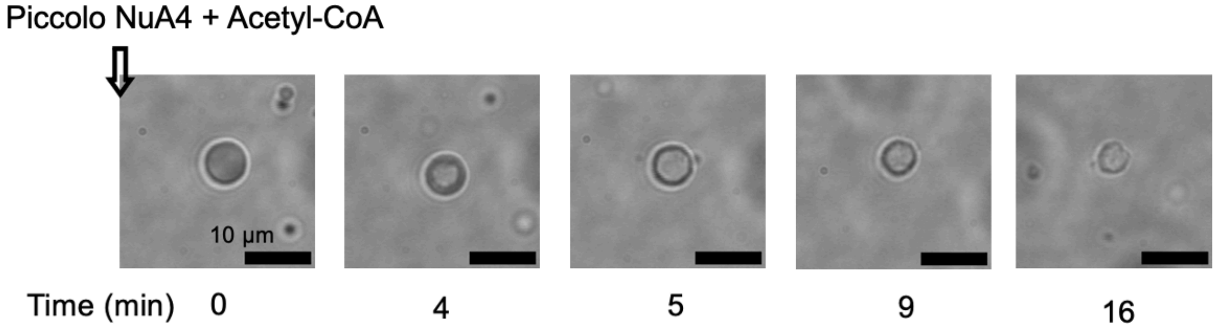

Supplementary Figure 8: Changes in the state and shape of a droplet upon treatment with Piccolo NuA4

Upon acetylation of H4 with Piccolo NuA4, droplets start showing crumbling from the inside, loss of the round outer fringe, and shrinkage of the droplet size within 10 – 15 minutes.

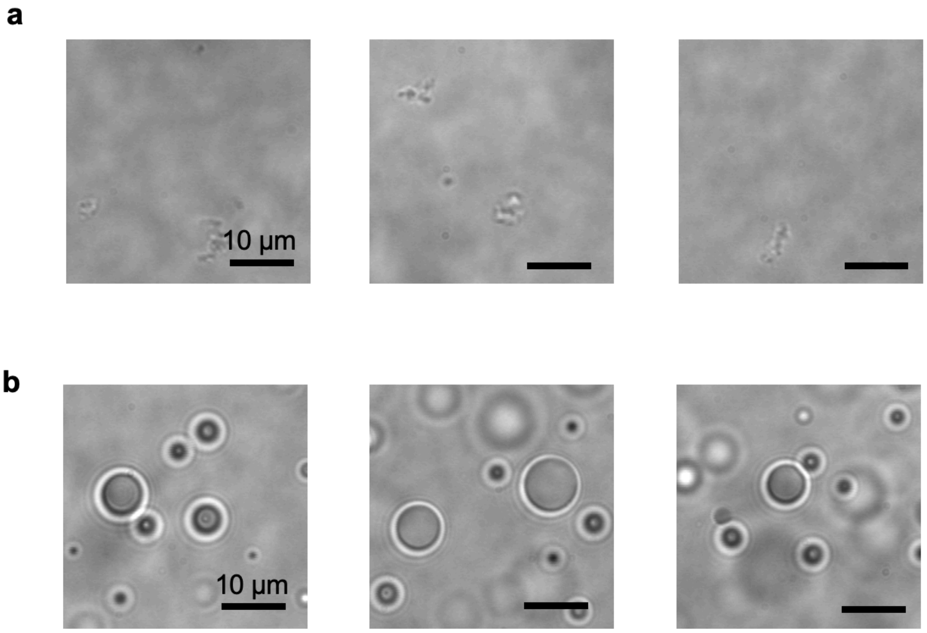

Supplementary Figure 9: Additional images of aggregates and droplets treated with Nap1

Additional microscopic images of **a** aggregates made of gH3 arrays that are dissolved by Nap1 (nucleosome:Nap1 = 1:2) after 20 min incubation and **b** droplets made of WT arrays 30 min after incubation with Nap1 (1:8).

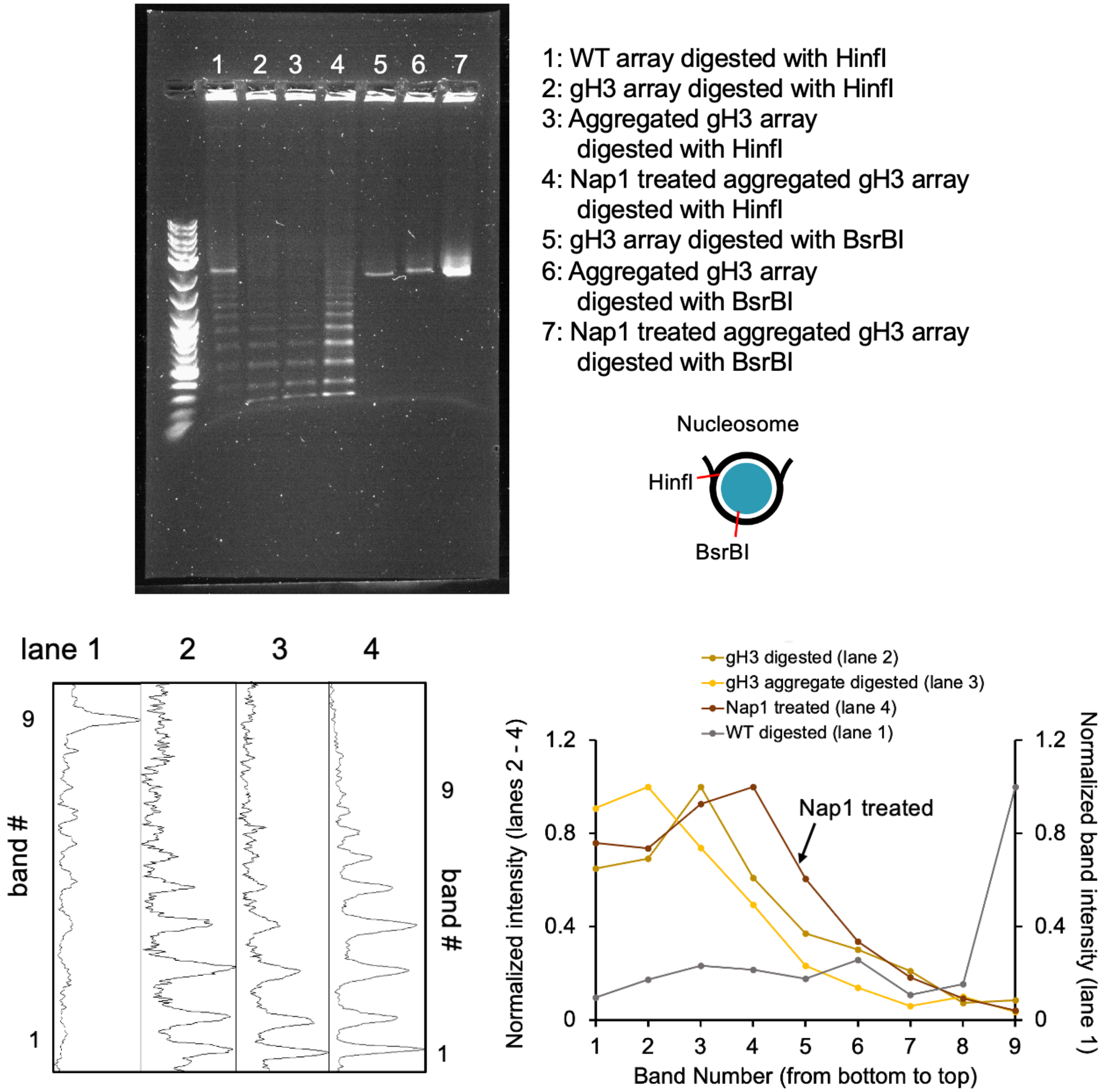

Supplementary Figure 10: Enzyme digestion assay to show the extent of nucleosome unwrapping in gH3 arrays

Nucleosome arrays (WT and gH3) were digested with HinfI and BsrBI to examine the extent of nucleosome unwrapping in gH3 arrays. The 601 nucleosome sequence contains a HinfI restriction site at the 5th – 10th nt from the entry and a BsrBI restriction site at the 23rd to 28th nt

from the entry. The gel shows partial opening of the nucleosome termini in gH3 arrays (lane 2). Aggregated gH3 arrays are digested more (lane 3 vs lane 2), supporting more unwrapping of nucleosomes in aggregates. Treatment of the aggregates with Nap1 makes the arrays digested much less, confirming that Nap1 mediated re-wrapping of the nucleosomes. The band intensities were normalized with the highest intensity band on each lane. The samples were digested following the protocols provided by the manufacturer (New England Biolabs). Briefly, 50 µL of a sample containing 0.2 µg of arrays in 20 mM Tris-OAc (pH 7.9) buffer with 50 mM KOAc, 10 mM MgOAc, and 100 µg/mL albumin was incubated with 2 units of HinfI or BsrBI for 1 hour at 37 ^{o}C with occasional mixing, followed by heat inactivation at 80 ^{o}C for 20 minutes. Upon heat inactivation, the samples were thoroughly mixed and loaded to an agarose gel.

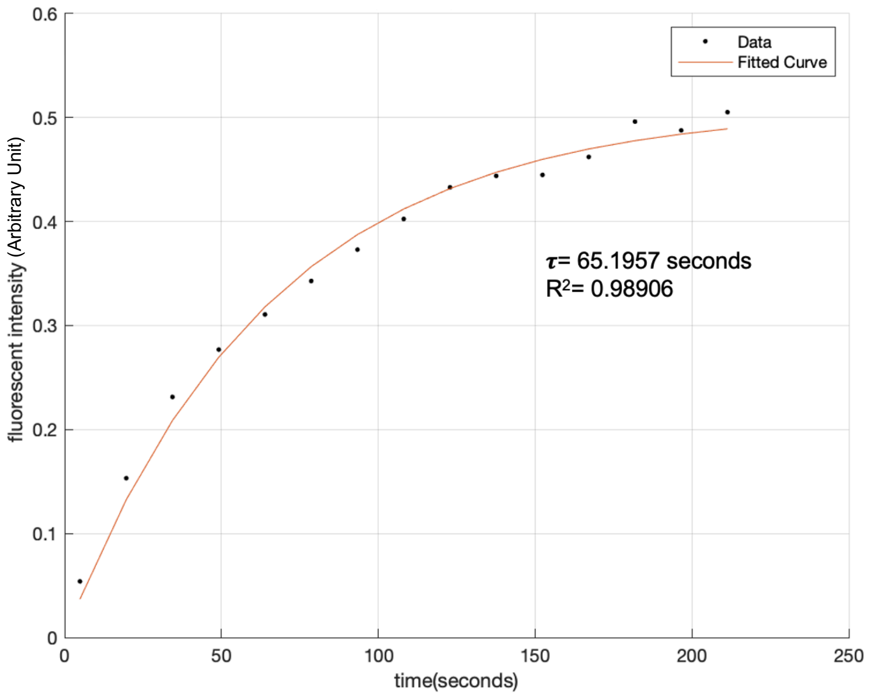

Supplementary Figure 11: Fluorescence recovery after photobleaching (FRAP) measurement

A typical fluorescence recovery curve after photobleaching within a droplet (WT nucleosome arrays, labeled at H4 E64C with Alexa Fluor 647) shows fitting with an exponential recovery function described in Methods.

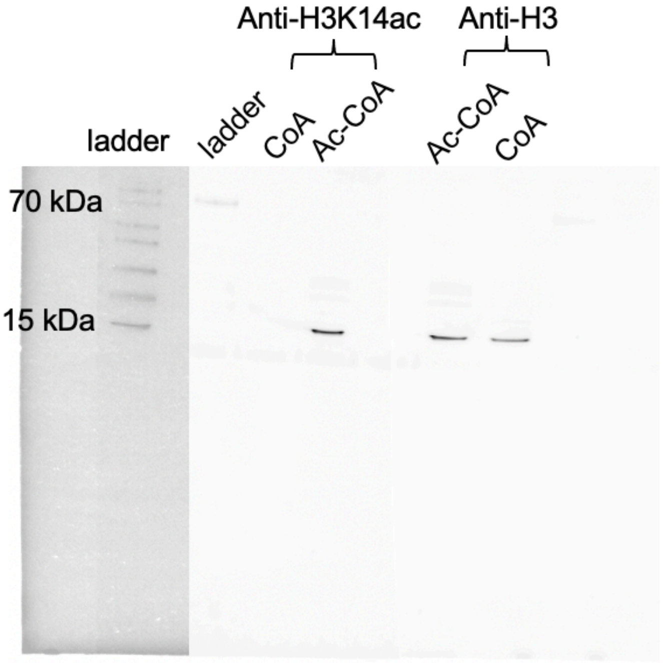

Supplementary Figure 12: Western blot verifying acetylation of histone H3 by Ada2/Ada3/Gcn5 in nucleosome arrays contained in condensates

Condensates formed with 200 nM WT arrays at 150 mM NaCl were incubated with 1 μM Ada2/Ada3/Gcn5 for 30 minutes in the presence of 1 mM CoA or acetyl-CoA (Ac-CoA), after which the condensates were sedimented by 10 min centrifugation at 2,000 x g. A bottom fraction of the sedimented sample was taken for a subsequent western blotting assay. A protein ladder and chromatin samples were first run through an SDS-PAGE (15 % acrylamide) gel, followed by transfer to a low fluorescence PVDF membrane. The membrane was cut into two pieces and each piece was incubated with a primary antibody (Anti-H3K14ac, H3K14ac polyclonal antibody from ThermoFisher Scientific (Cat# 720094) or Anti-H3, H3 polyclonal antibody from ThermoFisher Scientific (Cat# PA5-16183)). H3K14 is acetylated by Ada2/Ada3/Gcn5 and its anti-body has been employed to detect Ada2/Ada3/Gcn5 dependent acetylation (Haile et al. BBA – Gene Regulatory Mechanisms 1866 (2023) 194929). The membrane pieces were subsequently incubated with a secondary antibody labeled with Cy3. After rinsing, the membrane pieces were imaged side by side with a Typhoon biomolecular imager (Amersham). The darker ladder image

on the left was taken with a gel imager (GelDoc Go from Bio-Rad) in a white-epi mode and overlayed for comparison. The 70 kDa marker in the ladder (PageRuler^{TM} pre-stained protein ladder from ThermoFisher Scientific, Cat# 26616) has a weak fluorescence with Cy3 excitation and was used as a guide to overlay the two images. The final image clearly shows strong acetylation of the arrays in the condensates by Ada2/Ada3/Gcn5 only in the presence of Ac-CoA. Similar results were obtained with at least three independent experiments.

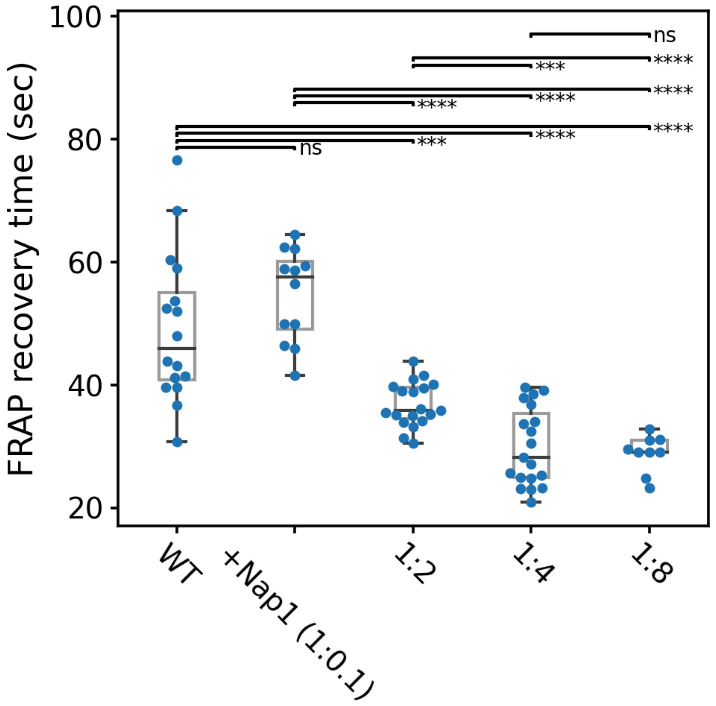

Supplementary Figure 13: Facilitated FRAP recovery of nucleosome arrays by Nap1 at varying ratios of nucleosome:Nap1 ratios

FRAP recovery times of WT nucleosome arrays labeled at H2A-H2B in the presence of Nap1 at varying nucleosome:Nap1 ratios show a significant change at 1:2, 1:4, and 1:8 ratios, but not at 1:0.1 (catalytic amount of Nap1). The results also show that the effect plateaus at 1:4, supporting the role for Nap1 in facilitating transient and repetitive nucleosome disassembly/reassembly in the H2A-H2B region, instead of disassembling nucleosomes completely, which should work only with a catalytic amount of Nap1. Sample sizes are n = 16, 12, 19, 19, and 9 for WT, +Nap1(1:0.1), 1:2, 1:4, and 1:8, respectively. Box plot elements are center line (median); box limits (upper and lower quartiles); whiskers (1.5x interquartile range). The significances shown are from two-sided student's t-test (*: p ≤0.05, **: p≤0.01, ***: p≤0.001, ****: p≤0.0001, ns: not significant). The p-values for comparisons of WT with +Nap1(1:01), with 1:2, with 1:4, and with 1:8 are 0.154, 0.00112, 1.11×10^{-5}, and 5.95×10^{-6}, respectively, those of +Nap1(1:0.01) with 1:2, with 1:4, and with 1:8 are 2.15×10^{-6}, 7.20×10^{-9}, and 1.73×10^{-8}, respectively, those of 1:2 with 1:4 and with 1:8 are 0.000314 and 7.79×10^{-6}, respectively, and that of 1:4 with 1:8 is 0.540. Source data are provided as a Source Data file.

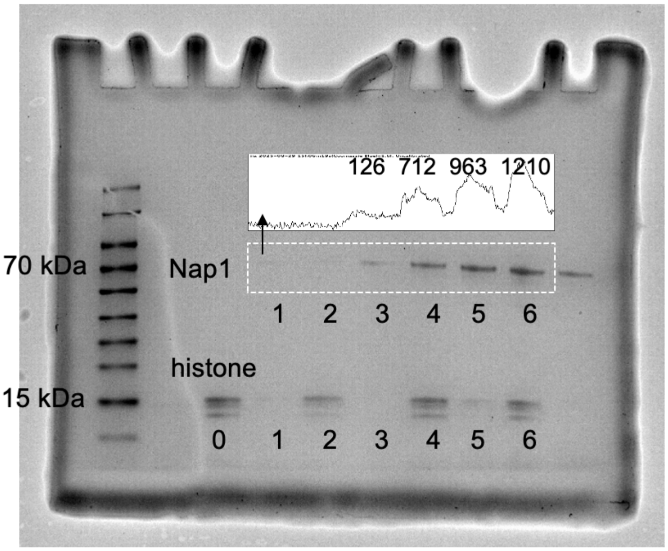

	Nucleosome:Nap1	layer
1:	1:2	supernatant
2:	1:2	condensates
3:	1:4	supernatant
4:	1:4	condensates
5:	1:8	supernatant
6:	1:8	condensates
0:	control (histone octamer only)	

Supplementary Figure 14: SDS-PAGE analysis to compare the amounts of Nap1 contained in the condensed and non-condensed phases

Droplets formed with WT arrays were sedimented by centrifugation (2,000 x g for 10 minutes), followed by an SDS-PAGE analysis to show the protein contents in the condensed (bottom fraction after centrifugation) and the non-condensed (supernatant) phases. The image clearly shows enriched Nap1 in the condensed phase at 1:4 and 1:8 nucleosome:Nap1 ratios. The bands are not detectable at 1:2 in either phase. It is tempting to propose that the enrichment is maximum at 1:4 as the difference in the Nap1 abundance between the two phases at 1:8 is not as large, suggesting saturation of the Nap1 enrichment at 1:4. Of note, the effect of Nap1 in enhancing H2A-H2B dynamics in condensates also saturates at 1:4 (Supplementary Figure 13), confirming that Nap1 interacts mainly with H2A-H2B in nucleosomes.

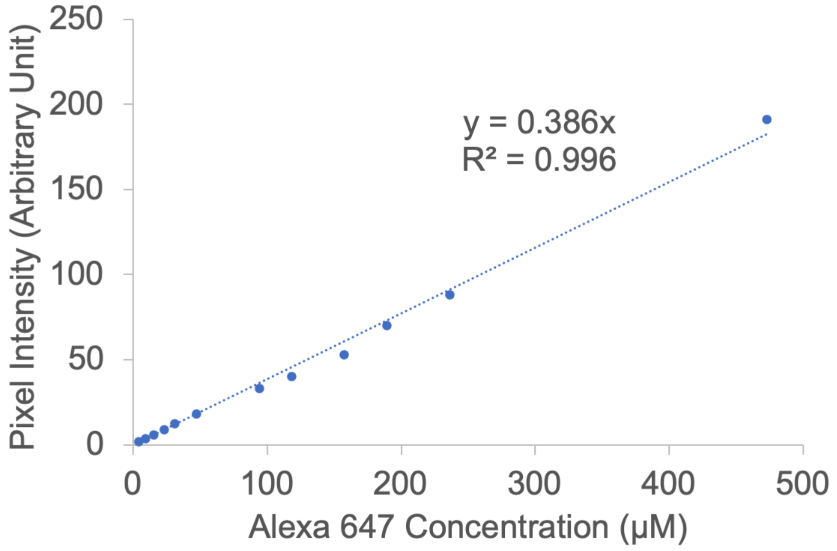

Supplementary Figure 15: Nucleosome concentration calibration

Varying concentrations of the same fluorophore used to label histone (Alexa Fluor™ 647) were imaged with the same microscope (LSM880, Zeiss) used to carry out the FRAP measurements and the concentration measurements of nucleosomes within droplets. The pixel counts from a random region of interest at each Alexa Fluor 647 concentration were averaged to obtain the pixel intensity within the precision allowed by 3 significant figures. This calibration curve was used to convert the intensities of the 16 central pixels of a chromatin droplet confocal image to the nucleosome concentrations. The average value is assigned as the nucleosome concentration of the droplet. The nucleosome concentration was divided by 2 to give the final concentration shown in Fig. 4b as one nucleosome contains two Alexa Fluor 647 fluorophores labeled at two histone H4 or H2B molecules.

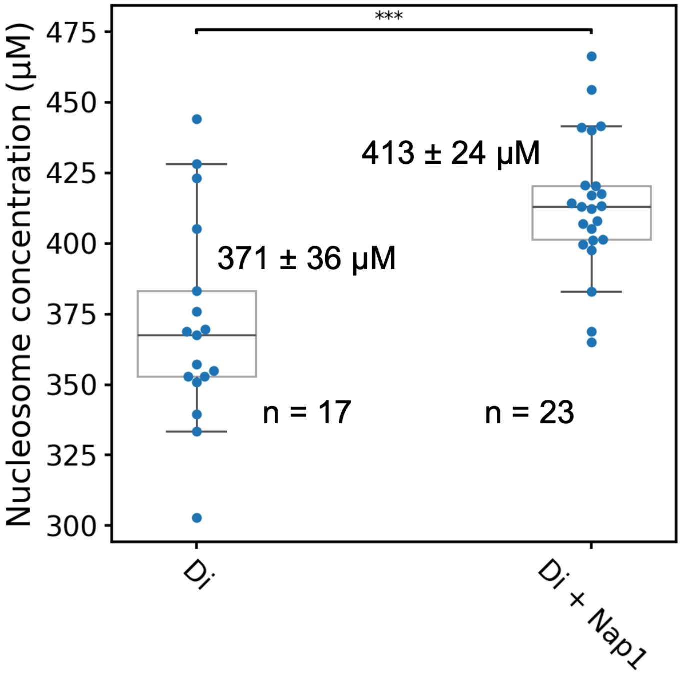

Supplementary Figure 16: Di-labeled nucleosome concentration increase in the presence of Nap1

Di-labeled array condensates show a higher nucleosome concentration in the presence of Nap1 according to the student's t-test (one-sided, ***: $p = 0.000156$). Box plot elements are center line (median); box limits (upper and lower quartiles); whiskers (1.5x interquartile range). The concentrations were measured from confocal images as described in the main text and Supplementary Figure 15. Sample sizes (n) are 17 and 23 respectively for the cases of without and with Nap1. Source data are provided as a Source Data file.

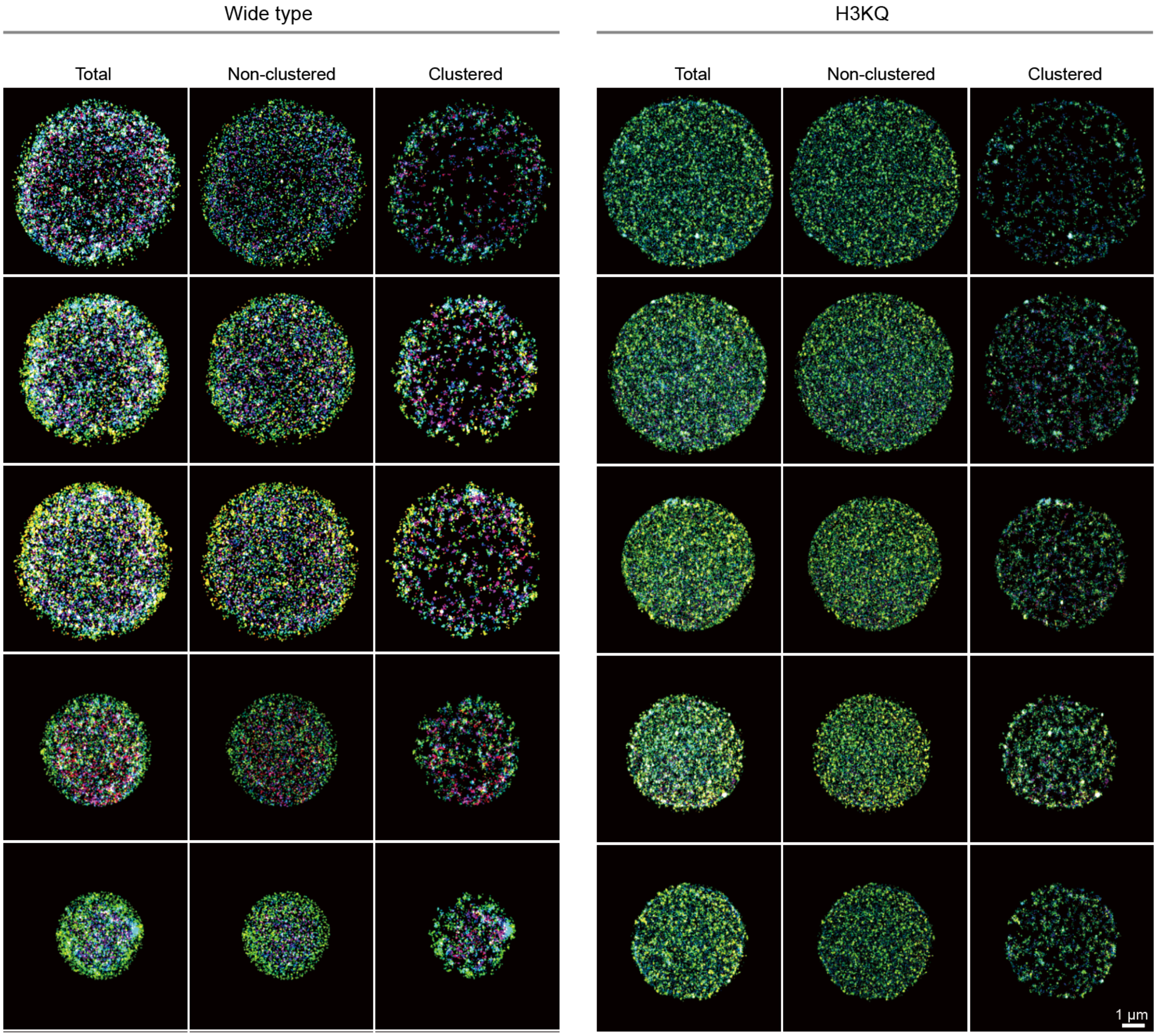

Supplementary Figure 17: Additional STORM images of droplets

Five additional STORM images of droplets of varying sizes made of WT arrays (left) and H3KQ arrays (right) clearly show both clustered (relatively immobile on the time scale of 10 sec) and non-clustered (mobile) fractions of nucleosome arrays that span the entirety of the droplet interior.

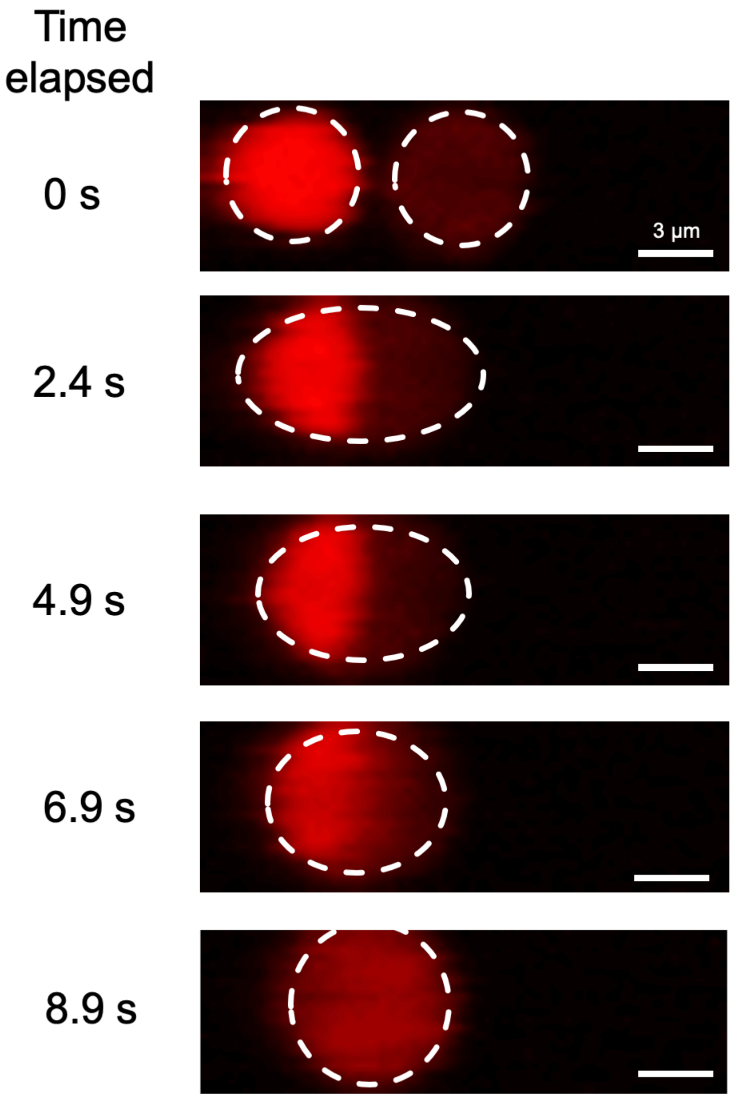

Supplementary Figure 18: Droplets showing immobile array fractions gradually mixing after fusion

One droplet formed with fluorescently labeled nucleosome arrays is fused with another droplet that is not fluorescent. During fusion, the content of the droplets start mixing >5 seconds after fusion, supporting relatively immobile fraction of nucleosome arrays filling the entire interior of the droplets.

Supplementary Table 1: FRAP recovery data

The data used for the FRAP recovery charts in Fig. 4 is tabulated. The recovery fractions are listed in the "A" columns and the recovery times are listed in the "τ" columns. Each array column is sorted according to the shortest to the longest τ values.

WT Tet		WT Di		Nap1 Tet		Nap1 Di		H3KQ Tet		H3KQ Di		H3ac Tet		H3ac Di	
A	τ (sec)	A	τ (sec)	A	τ (sec)	A	τ (sec)	A	τ (sec)	A	τ (sec)	A	τ (sec)	A	τ (sec)
0.389	43.1	0.536	30.8	0.593	22.7	0.487	16.3	0.543	13.0	0.366	10.4	0.492	29.9	0.501	24.1
0.501	44.3	0.567	36.7	0.594	24.5	0.597	17.1	0.469	13.5	0.429	11.8	0.563	31.8	0.541	25.5
0.574	48.9	0.541	39.6	0.501	25.6	0.504	17.8	0.476	15.2	0.551	13.5	0.576	32.1	0.553	26.4
0.409	51.8	0.626	39.7	0.566	26.7	0.507	19.3	0.459	15.7	0.539	14.0	0.497	32.7	0.523	29.4
0.529	52.2	0.564	41.3	0.540	26.7	0.526	19.4	0.494	15.7	0.551	14.5	0.525	33.9	0.513	31.2
0.478	59.4	0.597	41.4	0.519	27.1	0.526	19.5	0.488	17.5	0.507	15.6	0.548	37.7	0.497	31.7
0.493	60.0	0.656	43.2	0.426	27.5	0.545	19.8	0.358	22.4	0.501	17.3	0.472	37.9	0.530	35.1
0.512	60.8	0.458	43.8	0.427	27.6	0.535	21.5	0.380	23.1	0.487	17.8	0.503	38.5	0.529	35.6
0.509	65.0	0.688	48.0	0.525	27.6	0.539	22.2	0.448	25.5	0.561	17.9	0.527	46.3		
0.484	69.3	0.592	52.0	0.588	27.9	0.558	22.6			0.552	18.3				
0.505	81.2	0.552	52.5	0.570	32.4	0.440	24.2			0.535	18.6				
0.594	101.0	0.636	53.7	0.676	32.9	0.524	26.6			0.535	18.9				
		0.576	59.1	0.554	33.6	0.529	28.2			0.563	19.8				
		0.492	60.4	0.611	33.7	0.420	29.1			0.442	20.8				
		0.649	68.4	0.644	36.8	0.524	29.7			0.613	20.9				
		0.674	76.6	0.606	37.2					0.574	22.4				
				0.521	39.4					0.510	22.9				
